# Effect of iodoacetic acid on the reproductive system of male mice

**DOI:** 10.3389/fphar.2022.958204

**Published:** 2022-08-26

**Authors:** Yun Liang, Xinshuang Huang, Li Fang, Mingjie Wang, Chunxiao Yu, Qingbo Guan

**Affiliations:** ^1^ Shandong University of Traditional Chinese Medicine, Jinan, China; ^2^ Shandong Provincial Hospital, Shandong University, Jinan, China; ^3^ Shandong Clinical Research Center of Diabetes and Metabolic Diseases, Shandong Provincial Hospital, Jinan, China; ^4^ Shandong Laboratory of Endocrinology and Lipid Metabolism, Shandong Provincial Hospital, Jinan, China; ^5^ Shandong Prevention and Control Engineering Laboratory of Endocrine and Metabolic Diseases, Shandong Provincial Hospital, Jinan, China; ^6^ Shandong Provincial Hospital, Shandong University, Jinan, China; ^7^ Department of Endocrinology, Affiliated Hospital of Inner Mongolia Medical University, Inner Mongolia Medical University, Inner Mongolia, China

**Keywords:** iodoacetic acid, testis, sperm, testosterone, γH2AX, SRB1

## Abstract

Iodoacetic acid (IAA) is one of the most common water disinfection byproducts (DBPs). Humans and animals are widely and continuously exposed to it. Many species of water DBPs are harmful to the reproductive system of organisms. Nevertheless, the potential effects of IAA exposure on testosterone and spermatogenesis *in vivo* remain ambiguous. Spermatogenous cells are the site of spermatogenesis, Leydig cells are the site of testosterone synthesis, and Sertoli cells build the blood–testis barrier (BTB), providing a stable environment for the aforementioned important physiological functions in testicular tissue. Therefore, we observed the effects of IAA on spermatogenic cells, Leydig cells, and Sertoli cells in the testis. In this study, we found that oral administration of IAA (35 mg/kg body weight per day for 28 days) in male mice increased serum LH levels and reduced sperm motility, affecting average path velocity and straight line velocity of sperm. In addition, IAA promoted the expression of γH2AX, a marker for DNA double-strand breaks. Moreover, IAA downregulated the protein expression of the scavenger receptor class B type 1 (SRB1), and decreased lipid droplet transport into Leydig cells, which reduced the storage of testosterone synthesis raw materials and might cause a drop in testosterone production. Furthermore, IAA did not affect the function of BTB. Thus, our results indicated that IAA exposure affected spermatogenesis and testosterone synthesis by inducing DNA damage and reducing lipid droplet transport.

## Introduction

In recent years, male fertility has generally shown a downward trend (Agarwal et al., 2021), and the effect of endocrine disruptors is one of the reasons (Kahn et al., 2020). There are many types of endocrine disruptors, and water disinfection by-products (DBPs) are one of them. DBPs are chemical contaminants formed by the reaction between organic matter and disinfectants. Research proves that water DBPs disrupt ovarian function and spermatogenesis, and produce adverse reproductive outcomes (Gonsioroski et al., 2020a) because many species of these are cytotoxic, neurotoxic, genotoxic, carcinogenic, and teratogenic (Kali et al., 2021).

Haloacetic acid disinfection by-products (HAA-DBPs) are a class of water DBPs. Iodoacetic acid (IAA) is one type of HAA-DBPs. It is also used in the research of organic synthesis, dye industry, and plant resources, where people are exposed to a large dose of poisoning environment. Research has shown that IAA is the most genotoxic of all HAA-DBPs studied to date (Dong et al., 2019), which was more cytotoxic and genotoxic than their chlorinated and brominated analogues in primary human lymphocytes (Escobar-Hoyos et al., 2013). After inoculation into BALB/C nude mice, IAA transformed NIH3T3 cells into tumorigenic lines and formed aggressive fibro sarcomas, which demonstrated that it had a biological activity consistent with a carcinogen (Wei et al., 2013). IAA potentially disrupted the thyroid endocrine system by down-regulating the mRNA and protein expression levels of the thyrotropin receptor (TSHR) and the sodium/iodide symporter (NIS) (Xia et al., 2018). Moreover, IAA reduced cell viability significantly in the mouse primary hepatocytes (Wang et al., 2018).

For the adult female mice, IAA, as a hypothalamic–pituitary–gonadal axis toxicant, affected the pituitary directly and increased mRNA levels of kisspeptin (Kiss1) significantly in the arcuate nucleus (Gonzalez et al., 2021). Research demonstrated that IAA had reproductive and developmental toxicity (Long et al., 2021). Another study reported that IAA exhibited the most potent estrogenic activity (Kim et al., 2020). *In vivo*, IAA altered estrous cyclicity, ovarian gene expression, and estradiol levels in mice (Gonsioroski et al., 2021). Meanwhile, IAA inhibited follicle growth, decreased cell proliferation, and altered steroidogenesis *in vitro* (Jeong et al., 2016; Gonsioroski et al., 2020b). In the Comet assay, IAA resulted in DNA damage in sperm (Ali et al., 2014). However, the effect of IAA on the testis *in vivo* is not yet fully understood. Particularly, its effect on spermatogenesis and testosterone synthesis has not been elucidated.

To observe the effects of IAA on the male reproductive system and to test the mechanism, we constructed a model of IAA exposure by gavage and observed several common indicators of Leydig cells, Sertoli cells, and spermatogenic cells from the gene expression and protein levels. For spermatogenic cells, γH2AX, DAZL, and VASA were observed; for Leydig cells, scavenger receptor class B type 1 (SRB1), steroidogenic acute regulatory protein (STAR), P450 side-chain cleavage enzyme (CYP11A1), and cytochrome P450 17A1 (CYP17A1) were detected; and for Sertoli cells, WT-1, N-cadherin, and β-catenin were recorded. In addition, the levels of FSH, LH, and testosterone in the mouse serum were also determined.

## Materials and methods

### Materials and reagents

IAA was purchased from Sigma-Aldrich (Shanghai Warehouse, China). Antibodies for STAR (8449s) and CYP11A1 (14217s) were procured from Cell Signaling Technology, Inc. Antibody for CYP17A1 (14447-1-AP) was purchased from Wuhan SANYING Proteintech (Wuhan Warehouse, China). The other antibodies were obtained from Abcam (Shanghai, China) including DAZL (ab34139), VASA (ab13840), γH2AX (ab81299), SRB1 (ab217318), Wilms tumor-1 (WT-1) (ab89901), N-cadherin (ab18203), β-catenin (ab16051), and tubulin (ab7291).

### Animals and treatments

All animals used in the experiment were male 8 -week-old C57BL/6 mice, purchased from Vital River Laboratory Animal Technology Co., Ltd. (Beijing, China). They were randomly divided into two groups, with free access to water and food. After 1 week of acclimatization, the control group (8 mice) received treatment *via* gavage with a matching volume of double distilled H_2_O, and the experiment group (10 mice) was administered IAA at 35 mg/kg *via* oral gavage daily. The dosage and duration of IAA were referred to the previous literature (Xia et al., 2018). All mice were adapted to a 12-h light/dark cycle at 22–25°C. They were weighed twice a week, and doses were adjusted according to their bodyweight. After treatment for 28 days, all the mice were anesthetized, the blood was collected, and the caudal part of the epididymis was used for the evaluation of sperm parameters. Then the testis samples were removed quickly for the subsequent analyses. One testis was fixed in Bouin’s solution for the histopathological examination and immunofluorescent staining, and the other testis was frozen in liquid nitrogen and stored at −80°C until used for the assessment of mRNA and protein expression.

All animal experiments were approved by the Animal Ethics Committee of Shandong Provincial Hospital and performed according to the Shandong Provincial Hospital Animal Care and Use Committee.

### Sex hormone analysis

The level of luteinizing hormone (LH) in the serum was measured by the ELISA kit from CLOUD-CLONE CORP. (Wuhan, China). Testosterone and follicle stimulating hormone (FSH) in the serum were measured by the ELISA kit from Cusabio (Wuhan, China). The operating procedure was strictly in accordance with the kit instructions.

### Sperm quality

The cauda part of the right epididymis of each mouse was collected and cut into pieces with scissors to release sperms into an M199 culture medium (containing 1% BSA). The cultures were maintained in a humidified chamber (37°C, 5% CO_2_ incubator) for 5 min. A total of one hundred microliters of each sample was taken and diluted four times. Then thirty microliters of the sample were used for the following assay. Five pictures were taken per sample. Then the data were quantified by computer-aided sperm analysis (CASA, Hamilton-Thorne, Shanghai, China), and the appurtenant software was used to assess sperm concentration, motility, progressive, quantity, average path velocity, straight line velocity, and curvilinear velocity.

### Hematoxylin-eosin staining

The testis was immersed in Bouin’s fluid fixative for 24 h, transferred to 70% ethanol, and placed into an automated tissue processor for gradient ethanol dehydration. The samples were embedded in paraffin wax and cut into 3 μm sections. Then the testicular specimens were deparaffinized with xylene and ethanol, stained with hematoxylin and eosin, dehydrated, and mounted. Afterward, the morphological changes of the testis were observed under a microscope to complete the image collection and analysis.

### RNA extraction and real-time PCR

Total RNA was extracted from testicular tissue (10 mg) with TRIzol reagent (Takara, Tokyo, Japan) according to the manufacturer’s instructions. A Prime-Script RT Reagent Kit (Takara, Japan) was used to reverse-transcribe RNA into cDNA, and SYBR Premix Ex Taq (Takara, Japan) was used to perform a quantitative real-time polymerase chain reaction (PCR) utilizing a Thermal Cycler Dice Real-Time System (Takara, Japan). The program used to analyze the abundance of different genes was 95°C for 5 min, 40 cycles of 95°C for 10 s, 60°C for 10 s, and 72°C for 10 s, followed by a melting curve from 95 to 60°C, and cool to 37°C for 10 s. β-actin was employed as an endogenous control to normalize the data, and the 2-ΔΔCt calculation method was employed to analyze them. The qPCR primers used are listed in Supplementary Table S1.

### Western blotting

Mouse testicular fragments were lysed with ice-cold radioimmunoprecipitation assay buffer (RIPA buffer) supplemented with protease and phosphatase inhibitors (Shenergy Biocolor Bioscience & Technology Company, Shanghai, China), subjected to ultrasound pyrolysis, and centrifuged at 15,000 g for 15 min. Total protein was quantified using the BCA protein assay. After denaturation, proteins were separated by sodium dodecyl sulphate-polyacrylamide gel electrophoresis (SDS-PAGE) on 10%–12% polyacrylamide gels under reducing conditions. Separated proteins were electro transferred onto a polyvinylidene difluoride membrane (Millipore, Billerica, MA, United States) and blocked-in with 5% non-fat powdered milk containing 0.1% Tween 20 for 1 h. Next, to detect γH2AX, VASA, DAZL, SRB1, STAR, CYP17A1, CYP11A1, WT-1, N-cadherin, and β-catenin proteins (antibodies listed in Supplementary Table S2), the membrane was incubated with the respective primary antibodies at 4°C overnight. Thereafter, the membranes were washed briefly with TBST, incubated in the anti-rabbit or anti-mouse IgG secondary antibody conjugated to horseradish peroxidase (1:5000) for 1 h at room temperature, and visualized by a HyGLO HRP detection kit (Denville, NJ, United States). Protein expression levels were quantified with Fluor Chem Q SA software.

### Immunofluorescence

For fluorescent microscopy, paraffin sections of the testis were deparaffinized with xylene, hydrated with a series of alcohol solutions, and washed three times in phosphate-buffered saline (PBS). The specimens were immersed in EDTA antigen repair buffer and heated in a microwave oven for 25 min. Then they were cooled for 60 min, washed three times with PBS, and incubated with 5% donkey serum for 60 min at room temperature. Afterward, the slides were incubated with their respective primary antibodies overnight. The next day, the sections were washed three times with PBST and then incubated with FITC-conjugated donkey anti-rabbit IgG (1:1000; Thermo Fisher) for 1 h at room temperature. After a final wash in PBST, nuclei were stained with DAPI, the slides were visualized under a fluorescence microscope, and the photos were taken and analyzed.

### Statistical analysis

All data were analyzed with SPSS 25.0 and were expressed as the mean ± standard error of the mean (SEM). Outliers were removed by the ROUT’s test using GraphPad outlier calculator software. Means were compared using unpaired Student’s t-test for comparisons between two groups, and a two‐tailed value of *p* < 0.05 was considered statistically significant.

## Results

### Effects of iodoacetic acid on testicular morphology and hypothalamus pituitary gonadal axis hormones

**FIGURE 1:**
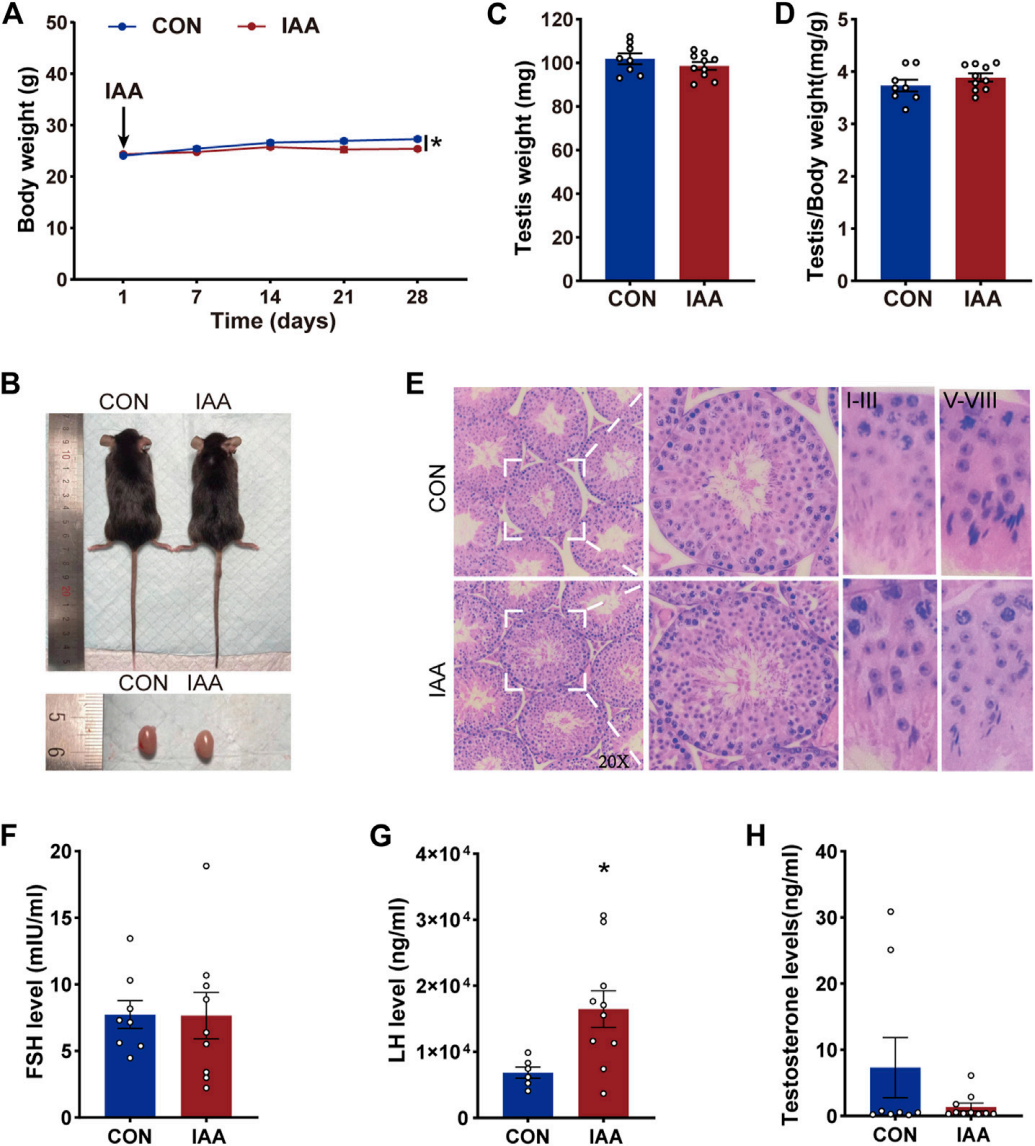
Effects of IAA exposure on general condition and serum hormone levels in mice. During the IAA exposure, the body weights of the mice were recorded twice a week. Only weekly values are shown in the graph **(A)**. Pictures of mice and testis **(B)**, testicular weight **(C)**, and the relative weight of testis **(D)** between the two groups were recorded after sacrifice. Hematoxylin-eosin (H&E) sections of testicular tissue of mice were also recorded and the pathological morphology was observed **(E)**. After IAA exposure, serum FSH, LH, and testosterone levels of mice were detected by ELISA **(F–H)**. Data are presented as the mean ± SEM. * Significant differences compared to control (*p* < 0.05).

Compared with the control group, a slight decrease in body weight was observed after the IAA exposure for 28 days (Figures 1A,B). The relative weight of testis was calculated by testes weight (mg)/body weight (g), but there were no significant reductions in the testis weight and its relative weight between two groups (Figures 1C,D). As shown in Figure 1E, no evidence of atrophy and vacuoles was seen in the experimental group. The testis tissue presented a normal testicular morphology and complete seminiferous tubules, and also possessed different stages of spermatogonium, spermatocyte, and spermatozoa in the seminiferous tubules. IAA did not alter the pathology and morphology of the testicular tissues. To clarify the effect of IAA exposure on sex hormones, serum FSH, LH, and testosterone levels were detected. There was no significant difference in the FSH level between the two groups (Figure 1F). However, a significant increase in the LH level was observed versus control (*p* = 0.020, Figure 1G). Although there was no significant reduction in the testosterone level, a descending trend was observed compared with the control group (*p* = 0.166, Figure 1H).

### Effects of iodoacetic acid exposure on sperm motility

To observe the effect of IAA exposure on sperm motility, various indicators of sperm motility and concentration were tested (as listed in Table 1). As observed in our results, there were no significant changes in the following sperm parameters, such as sperm concentration, motile, progressive, and curvilinear velocity (VCL). But the average path velocity (VAP) and straight line velocity (VSL) of sperm decreased significantly compared with the control group. We also tested the sperm ratios as rapid, medium, slow, and static. There were no statistical differences in these indicators between the two groups; however, there was a clear increasing trend in the proportion of slow motile sperm.

**TABLE 1:** Sperm parameters.

	CON	IAA	*p* value
VAP (μm/s)	87.10 ± 4.40	80.65 ± 7.12	0.040*
VSL (μm/s)	63.94 ± 5.27	57.79 ± 5.91	0.035*
VCL (μm/s)	184.93 ± 9.50	177.69 ± 16.19	0.281
ALH (μm)	8.98 ± 0.51	9.03 ± 0.51	0.822
BCF (Hz)	36.09 ± 0.71	35.91 ± 1.21	0.719
LIN (%)	36.62 ± 2.92	35.40 ± 2.37	0.340
STR (%)	70.75 ± 3.45	69.70 ± 2.58	0.471
Concentration (M/ml)	9.63 ± 1.92	8.21 ± 1.69	0.116
Motile (%)	53.63 ± 9.50	56.00 ± 11.10	0.638
Progressive (%)	20.00 ± 4.57	17.80 ± 5.35	0.369
Rapid sperm (%)	11.63 ± 5.40	39.70 ± 6.57	0.621
Medium sperm (%)	41.13 ± 5.09	15.50 ± 8.06	0.262
Slow sperm (%)	0.65 ± 0.26	0.95 ± 0.41	0.097
Static sperm (%)	46.38 ± 9.50	44.00 ± 11.10	0.638

*Significant differences compared to the control (*p* < 0.05).

### Effects of iodoacetic acid exposure on markers of spermatogenic cells

**FIGURE 2:**
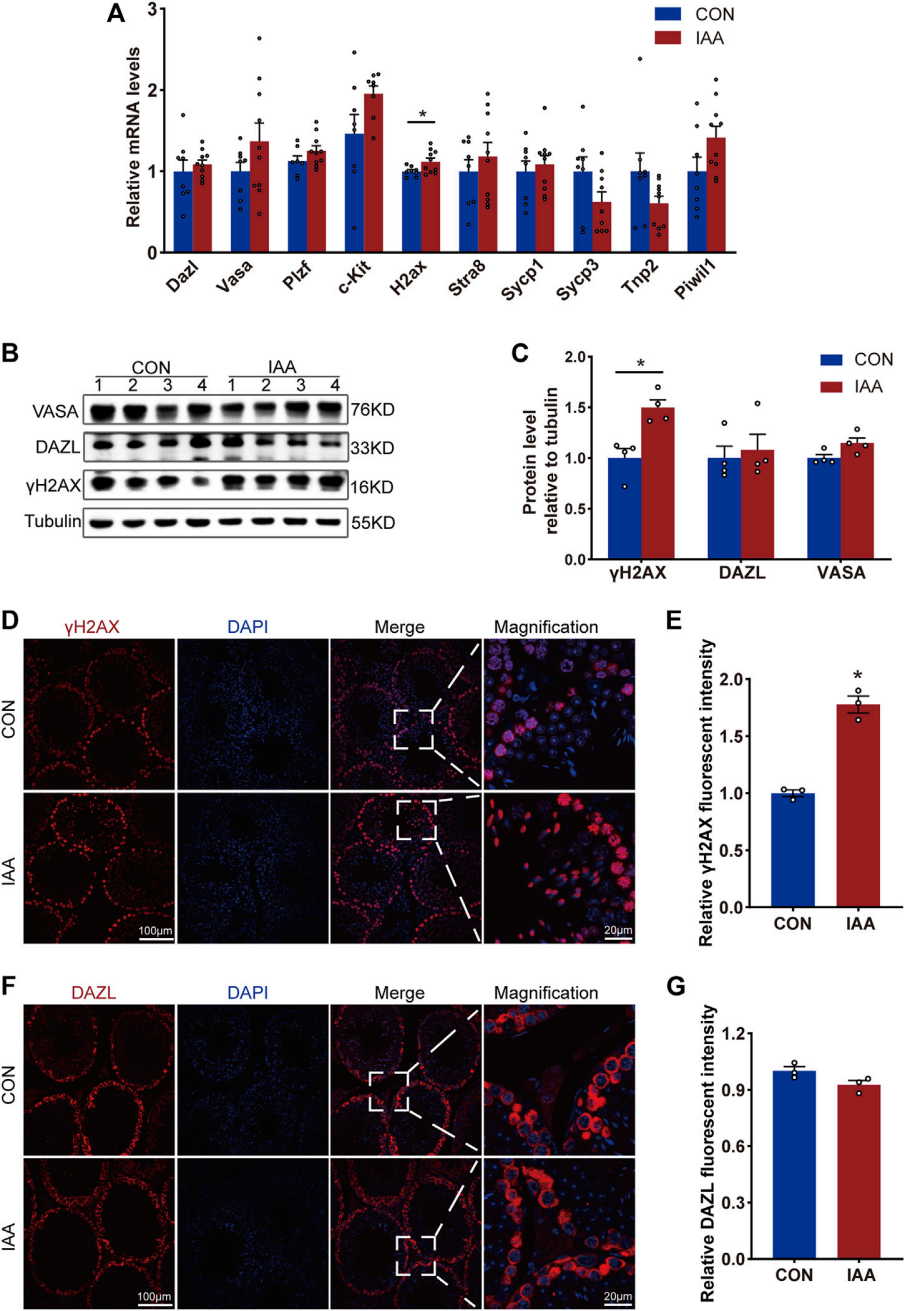
Changes of spermatogenesis-related indexes were observed from gene expression and protein level, respectively. The expression of the following genes, such as Dazl, Vasa, Plzf, c-Kit, H2ax, Stra8, Sycp1, Sycp3, Tnp2, and Piwil1, in the testis tissue of the two groups were detected by real-time PCR (qRT-PCR) technology **(A)**. Also, the changes of protein levels such as VASA, DAZL, and γH2AX between the two groups of mice were observed by Western Blot **(B,C)** and immunofluorescence staining experiments **(D–G)**. * Significant differences compared to the control (*p* < 0.05).

Spermatogenesis is an important physiological function of testicular tissue. To observe the effects of IAA exposure on spermatogenic cells, the gene expression and protein levels of relevant indicators during spermatogenesis were examined. As shown in Figure 2A, no significant changes were observed in the spermatogenesis indicators Dazl, Vasa, Plzf, c-Kit, Stra8, Sycp1, Sycp3, Tnp2, and Piwil1 at the mRNA level between the two groups. But IAA treatment significantly increased γH2AX expression compared to no treatment. All seminiferous tubules contain γH2AX spermatocytes at respective steps in development. Meanwhile, γH2AX is a marker of the DNA damage response and replication stress. Although IAA exposure did not change the protein levels of VASA and DAZL compared to the control (Figures 2B,C,F,G), the protein level of γH2AX was observed increasingly by Western Blot (WB) and immunofluorescence staining experiments (Figures 2B–E).

### Effects of iodoacetic acid on cholesterol transport and testosterone synthesis-related enzymes

**FIGURE 3:**
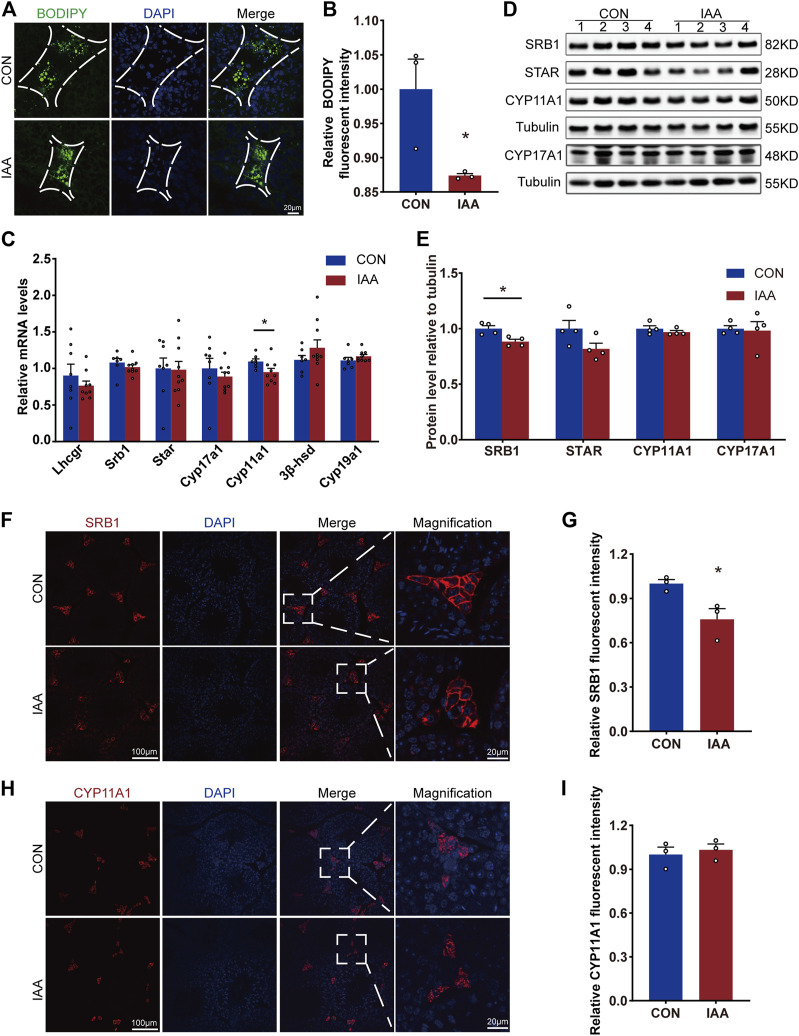
Lipid droplet storage in Leydig cells and changes in testosterone synthase from gene expression and protein levels were examined. Cholesterol storage in Leydig cells between the two groups was visualized by BODIPY staining **(A,B)**. The relative mRNA levels of the following indicators, Lhcgr, Srb1, Star, Cyp17a1, Cyp11a1, 3β-hsd, and Cyp19a1, were detected by qRT-PCR **(C)**. Also, the protein levels of cholesterol transporter SRB1 and testosterone synthase STAR, CYP11A1, and CYP17A1 were observed by Western Blot **(D,E)** and immunofluorescence staining experiments **(F–I)**. * Significant differences compared to the control (*p* < 0.05).

To evaluate the effect of IAA exposure on testosterone synthesis, changes in gene expression, protein levels of these enzymes and LDs were examined. The results demonstrated that IAA exposure decreased LD storage in Leydig cells (Figures 3A,B). SRB1, as a high-density lipoprotein receptor, is responsible for the transport of LDs. In our study, although the gene expression did not change between the two groups (Figure 3C), there was a decrease in the protein level (Figures 3D–G). Compared with the control group, a mild decrease in Cyp11a1 mRNA expression was observed, as shown in Figure 3C. However, its protein level showed no significant difference between the two groups (Figures 3D,E,H,I). The steroidogenic acute regulatory (STAR), as a key enzyme in testosterone synthesis, tended to decrease in the experiment group, but the differences failed to reach statistical significance (Figures 3D,E). In the immunofluorescence staining experiment, it showed no difference *versus* control (Supplement Figures 1A,B). Also, no changes were observed in the gene expression of Cyp17a1, Cyp19a1, 3β-hsd, and Lhcgr between the two groups (Figure 3C).

### Effects of iodoacetic acid on blood–testis barrier function in Sertoli cells

**FIGURE 4:**
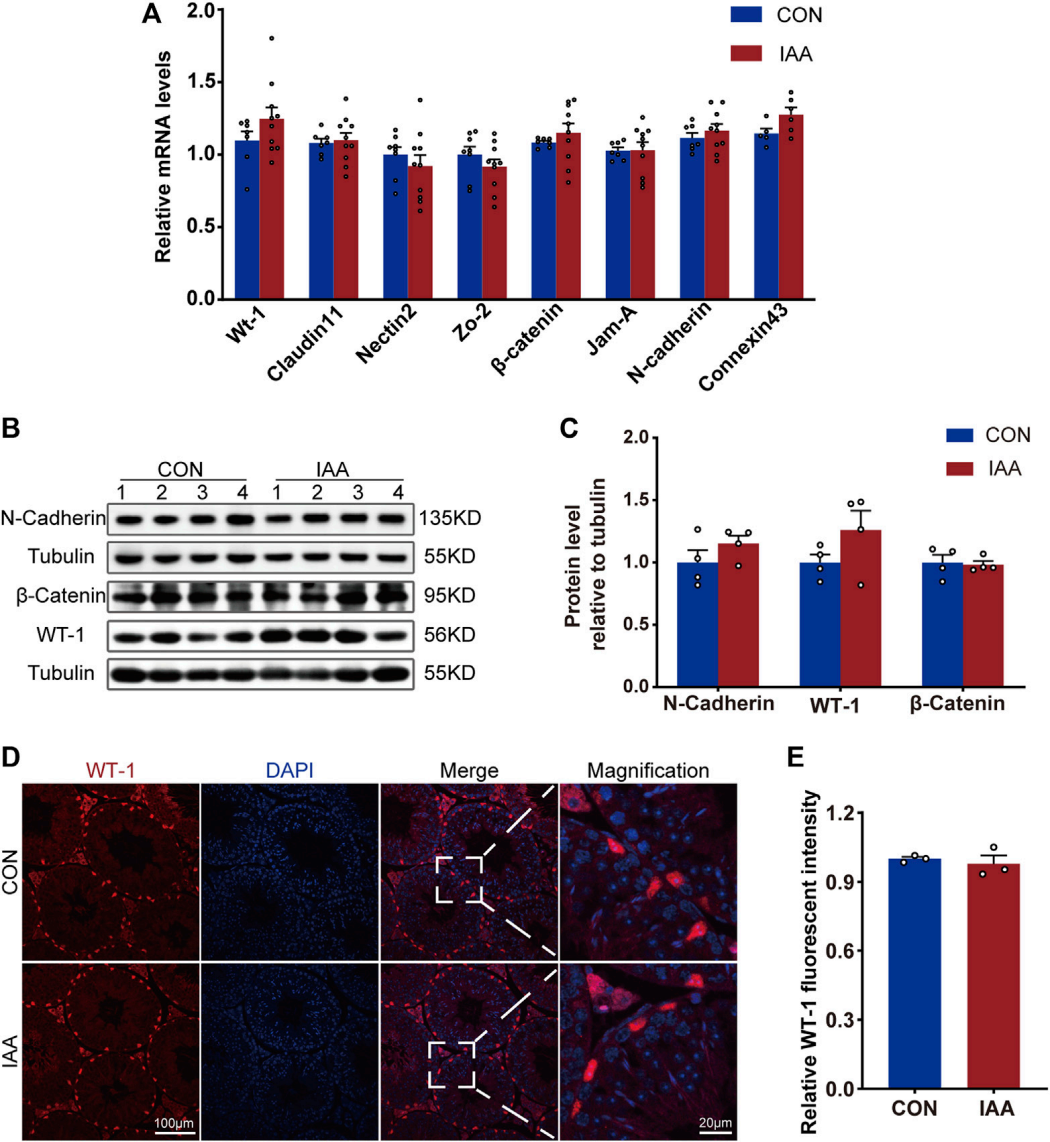
Changes of BTB-related indexes were detected from gene expression and protein level. Relative mRNA levels between the two groups, Wt-1, Claudin-11, Nectin-2, Zo-2, β-catenin, Jam-A, N-cadherin and Connexin-43, were observed **(A)**. The protein levels of WT-1, N-cadherin, and β-catenin were also observed by Western Blot **(B,C)** and immunofluorescence staining experiments **(D,E)**. * Significant differences compared to the control (*p* < 0.05).

To assess the effect of IAA exposure on Sertoli cells, changes in genes and proteins of WT-1 and some indicators of tight junctions were examined. As shown in Figure 4A, there were no differences at the genetic level between the two groups on Wt-1, Claudin-11, Nectin-2, Zo-2, β-catenin, Jam-A, N-cadherin, and Connexin-43. The protein levels of WT-1, N-cadherin, and β-catenin also did not perform a statistical difference compared with control (Figures 4B–E).

## Discussion

It is well known that endocrine disruptors have a direct impact on gonads and long-term reproductive health (Delbes et al., 2022). IAA has a negative influence on the female reproductive system, while the effects on the male reproductive system have not been systematically studied. From the perspective of the physiological structure of the testis, the male reproductive system mainly includes spermatogenic cells, Leydig cells, and Sertoli cells (Mäkelä et al., 2019). Therefore, we mainly observed the effect of IAA on its function from these three aspects. In our study, IAA exposure had no apparent effect on Sertoli cell function, but it exacerbated DNA damage in spermatogenic cells and reduced cholesterol storage in Leydig cells by reducing the protein expression of SRB1. In addition, IAA also affected sperm motility, slowing down VAP and VSL. Furthermore, it caused dysfunction of the hypothalamic–pituitary–gonadal axis, especially the increase in LH in serum.

We found that the body weight in the experimental group showed a slight decrease. This may be mainly due to the effect of IAA on gastric acid secretion (Cheng et al., 2001), which finally caused the mice to lose weight. To observe the effect of IAA on sex hormones *in vivo*, we measured the levels of FSH, LH, and testosterone in the serum. In our study, FSH did not change compared to the control group, but LH showed a significant increase *versus* control. Research showed that in adult female mice, IAA did not alter LHβ expression, while it reduced FSHβ-positive cell number and FSHβ mRNA expression at a dose of 10 mg/kg *in vivo* (Gonzalez et al., 2021). The inconsistent results might be due to the difference in dosage and time of medication. Given that testosterone varies widely between individuals, we did not remove outliers. Although there was no difference in testosterone between the two groups, there was a descending trend in the experimental group. We inferred that IAA exposure caused a decrease in testosterone but increased LH through negative feedback, thus maintaining normal levels of testosterone.

To observe the effect of IAA on sperm, we examined changes in relevant sperm parameters, such as sperm concentration, motility, progressiveness, VAP, VSL, VCL, ALH, and BCF. Among them, VAP and VSL showed a significant decrease compared to the control group. We deduced that was because IAA inhibited the glyceraldehyde-3-phosphate dehydrogenase (GAPDH), which was the key target of IAA (Hall et al., 2020) and one of the key enzymes in glycolysis, reduced cellular ATP levels (Dad et al., 2018), and ultimately slowed down sperm motility.

Sperm production is one of the most important functions of the testis. Spermatogenesis is a complex developmental process that consists of three stages: mitosis, meiosis, and spermiogenesis (Zhao J. et al., 2021). To observe the effect of IAA on spermatogenesis, protein levels of DAZL, VASA, γH2AX, and related gene expression changes were detected. Phosphorylated histone H2AX (γH2AX) is a hallmark of chromatin remodeling in male meiosis (Abe et al., 2020). Meanwhile, it is a sensitive marker for DNA double-strand breaks (DSBs) (Arnould et al., 2021), and a number of studies have regarded it as a marker of DNA damage (Guo et al., 2021; Zhang et al., 2021; Wang et al., 2022). Increased expression of γH2AX levels at the gene expression and protein level are detected, indicating that DNA damage is exacerbated after IAA exposure. DAZL, which is crucial for normal spermatogenesis, plays an important role in primordial germ cell formation, and genetic loss of DAZL causes infertility in both sexes of mice (Li et al., 2019). In our study, changes in DAZL gene and protein levels were not observed. VASA (DDX4/MVH), as the hallmark of meiotic cells at the stage from pachytene spermatocytes to round spermatids, is essential for male gametogenesis (Lin et al., 2017). It also showed no difference between the two groups in our study. Plzf and Kit are essential in maintaining spermatogonial stem cell proliferation (Mao et al., 2017; Yu et al., 2022). Stra8 (Sun et al., 2022), Sycp1 and Sycp3 (Dunce et al., 2018), and Tnp2 (Gustafson et al., 2020) play important roles in different stages of meiosis. Also, knockout of the Piwi gene in mice and human causes sterility (Gou et al., 2017). Fortunately, with the exception of H2AX, IAA exposure did not cause changes in the other genes described previously.

The second important function of the testis is testosterone biosynthesis. In order to observe the effect of IAA on testosterone, we focused on the changes in cholesterol and key enzymes in cholesterol transport and testosterone synthesis. SRB1 is a cell surface HDL receptor that mediates HDL-cholesteryl ester (CE) uptake, and it promotes the transport of CEs to Leydig cells and stores them as lipid droplets (LDs), which are used for the synthesis of testosterone in testis tissue (Shen et al., 2018). In our study, the content of cholesterol in Leydig cells decreased in the IAA-exposed group. This was because IAA affected LD transport by reducing the protein expression of SRB1, although IAA did not alter the gene expression of it. We infer that IAA causes changes in SRB1 protein levels through post-transcriptional translation or epigenetic modification, which, of course, requires further experimental validation. At the same time, we also detected a series of key enzymes in testosterone synthesis, such as STAR, CYP11A1, CYP17A1, and 3β-HSD. Except for the decrease of Cyp11a1 at the gene level, other indicators did not show significant differences both in gene expression and at the protein level. However, STAR, one of the most critical enzymes regulating testosterone synthesis (Zhao L. et al., 2021), showed a significant downward trend at the protein level, which further corroborated our conclusion that testosterone had declined during the medication. In Leydig cells, LHCGR acts as an LH receptor to sense the control of testosterone synthesis by the hypothalamic–pituitary–testicular axis (Holota et al., 2020). Gene expression of Lhcgr did not change between the two groups. In addition, the gene level of Cyp19a1, a key enzyme that converts testosterone to estrogen, was examined, and did not change compared to control. This means IAA does not influence the conversion of testosterone to estrogen in male mice.

An important function of Sertoli cells is to form the BTB, which forms the microenvironment for spermatogenesis and protects spermatogenic cells from autoimmune reactions. To assess the function of the BTB, we examined the expression of WT-1, N-cadherin, and β-catenin. WT-1, expressed in all Sertoli cells, is essential for germ cell survival and spermatogenesis (Gupta et al., 2021). N-Cadherin is a protein with greater connection flexibility, which is highly expressed in testis tissue (Verón et al., 2021). At the same time, in other tissues, it is often regarded as highly correlated with tissue migration, and is even used as one of the indicators of tumor aggressiveness (He et al., 2021). β-Catenin is also a characteristic protein of the BTB, and its abnormal accumulation can lead to impaired testicular junction integrity, which can lead to abnormal structures and functions of the BTB (Lei et al., 2021). In our study, the gene expression and protein levels of WT-1, N-cadherin, and β-catenin were not different between the two groups. As important components of tight junctions, such as claudin11 (She et al., 2021), Nectin-2 (Bronson et al., 2017), Zo-2 (Otani et al., 2019), Jam-A (Ebnet, 2017), and Connexin 43 (Liang et al., 2019), they all play a key role in maintaining the integrity of the BTB. Fortunately, there were no statistically significant differences in their gene levels.

In conclusion, in male mice, IAA reduced sperm motility, exacerbated DNA damage, and decreased storage of LDs, the raw material for testosterone synthesis. Of course, a deeper mechanism is needed in further research. At present, the adverse health consequences of endocrine disruptors have been known to all sectors of society (La Merrill et al., 2020), and various countries are also formulating some corresponding regulatory measures (Kassotis et al., 2020). However, little attention has been paid to water DBPs such as IAA. It not only affects the health of frontline workers producing such materials but also has long-term human contact in the form of water DBPs. Given that these endocrine disruptors have toxic effects on humans and even transmit their toxic effects to offspring in a genetic or epigenetic manner (Lombó and Herráez, 2021), laws and regulations on the control of them should be formulated as soon as possible. Also, we should try to find other healthier disinfection methods to replace chlorine-containing disinfectants to reduce their harm to the reproductive system and even human health.

## Data Availability

The original contributions presented in the study are included in the article/Supplementary Material; further inquiries can be directed to the corresponding authors.
