# Supplementary material for: Effect of iodoacetic acid on the reproductive system of male mice
Source: Front Pharmacol. 2022 Aug 26;13:958204. doi: 10.3389/fphar.2022.958204 (PMC9461136; doi:10.3389/fphar.2022.958204)
Supplement: Supplementary file 1 [file Table1.DOCX]

Supplementary Material

# Supplementary Figures and Tables

## Supplementary Figures


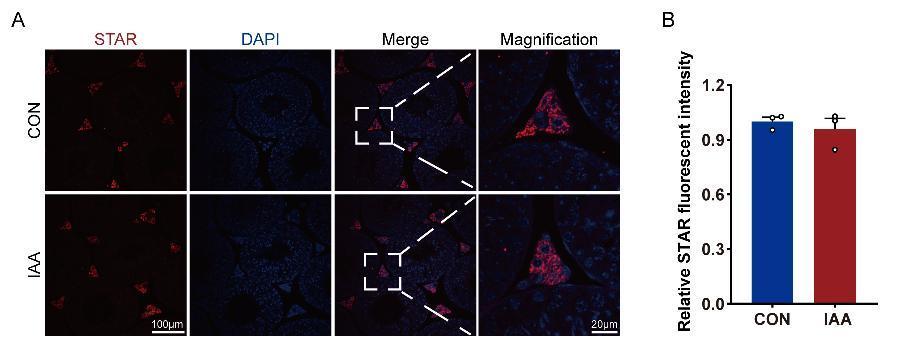


**Supplementary Figure1** The protein changes of STAR between the two groups were detected by immunofluorescence (A, B).


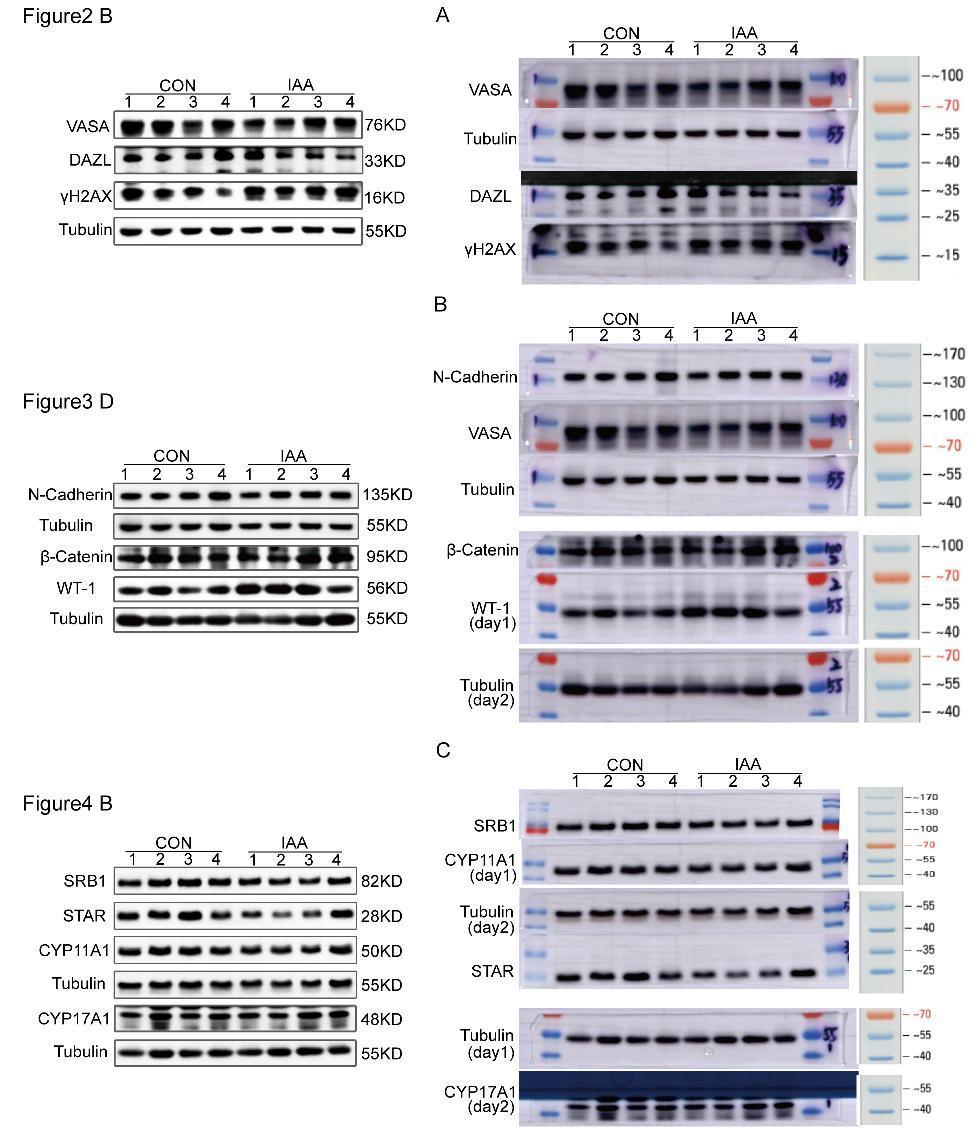


**Supplementary Figure2** The cropped images of blots are shown in figure 2B,3D,4B. And the full scan of the entire original gel(s) are shown in A, B, C above.

## Supplementary Tables

**Supplementary Table1**. List of Primers Used for qRT-PCR

| **Gene** | **Forward primer** | **Reverse primer** |
| --- | --- | --- |
| **Dazl** | ATGTCTGCCACAACTTCTGAG | CTGATTTCGGTTTCATCCATCCT |
| **Vasa** | GCTTCATCAGATATTGGCGAGT | GCTTGGAAAACCCTCTGCTT |
| **Plzf** | CTGCGGAAAACGGTTCCTG | GTGCCAGTATGGGTCTGTCT |
| **c-Kit** | CTCCCCCAACAGTGTATTCAC | TAGCCCGAAATCGCAAATCTT |
| **H2ax** | CGGTGGGCTTGAAGGTTAGT | ACTGGTATGAGGCCAGCAAC |
| **Stra8** | ACAACCTAAGGAAGGCAGTTTAC | GACCTCCTCTAAGCTGTTGGG |
| **Sycp1** | TGAGGGGAAGCTCACGGTT | CGAACAGTGTGAAGGGCTTTTG |
| **Sycp3** | AGCCAGTAACCAGAAAATTGAGC | CCACTGCTGCAACACATTCATA |
| **Tnp2** | TCACACCAGTAACCAGTGCAA | CCCTGAGCTACGCCTCTTAG |
| **Piwil1** | CAGCAACCTGGGTACATCCC | CCAAGGTCATGGAAGTCTCGG |
| **Lhcgr** | CGCCCGACTATCTCTCACCTA | GACAGATTGAGGAGGTTGTCAAA |
| **Srb1** | AAACAGGGAAGATCGAGCCAG | GGTCTGACCAAGCTATCAGGTT |
| **Star** | ATGTTCCTCGCTACGTTCAAG | CCCAGTGCTCTCCAGTTGAG |
| **Cyp17a1** | GCCCAAGTCAAAGACACCTAAT | GTACCCAGGCGAAGAGAATAGA |
| **Cyp11a1** | TGCTTGAGAGGCTGGAAGTTGA | CGGATTGCGGAGCTGGAGAT |
| **3β-hsd** | TATTCTCGGTTGTACGGGCAA | GTGCTACCTGTCAGTGTGACC |
| **Cyp19a1** | ATGTTCTTGGAAATGCTGAACCC | AGGACCTGGTATTGAAGACGAG |
| **Wt-1** | GAGAGCCAGCCTACCATCC | GGGTCCTCGTGTTTGAAGGAA |
| **Claudin11** | ATGGTAGCCACTTGCCTTCAG | AGTTCGTCCATTTTTCGGCAG |
| **Nectin2** | TCCTACGATCCAAAGACTCAGG | AGGTAGCATGAGACCTTGCTC |
| **Zo-2** | ATGGGAGCAGTACACCGTGA | TGACCACCCTGTCATTTTCTTG |
| **β-catenin** | ATGGAGCCGGACAGAAAAGC | CTTGCCACTCAGGGAAGGA |
| **Jam-A** | TCTCTTCACGTCTATGATCCTGG | TTTGATGGACTCGTTCTCGGG |
| **N-cadherin** | AGCGCAGTCTTACCGAAGG | TCGCTGCTTTCATACTGAACTTT |
| **Connexin43** | ACAGCGGTTGAGTCAGCTTG | GAGAGATGGGGAAGGACTTGT |
| **β-actin** | CTAAGGCCAACCGTGAAAAGA | CCAGAGGCATACAGGGACAAC |

**Supplementary Table2**. List of Antibodies Used for Western blotting and Immunofluorescence

| **Antibodies/Reagent** | **Source** | **Identifier** |
| --- | --- | --- |
| **BODIPY** | **Invitrogen** | **D3922** |
| **γH2AX** | **Abcam** | **ab81299** |
| **VASA** | **Abcam** | **ab13840** |
| **DAZL** | **Abcam** | **ab34139** |
| **SRB1** | **Abcam** | **ab217318** |
| **STAR** | **Cell Signaling Technology** | **8449s** |
| **CYP17A1** | **Wuhan SANYING** | **14447-1-AP** |
| **CYP11A1** | **Cell Signaling Technology** | **14217s** |
| **WT-1** | **Abcam** | **ab89901** |
| **N-Cadherin** | **Abcam** | **ab18203** |
| **β-Catenin** | **Abcam** | **ab16051** |
